# Screening for anti-*Leishmania* antibodies and *Leishmania* infections in kidney transplant recipients and donors from Brazil

**DOI:** 10.1590/S1678-9946202567067

**Published:** 2025-10-03

**Authors:** Gustavo Henrique Johanson, Maria Carmen Arroyo Sanchez, Regina Maia de Souza, Beatriz Julieta Celeste, Ruth Tamara Valencia-Portillo, Elias David, Ligia Camera Pierrotti, Valdir Sabbaga Amato

**Affiliations:** 1 Universidade de São Paulo, Faculdade de Medicina, Instituto de Medicina Tropical de São Paulo, Departamento de Infectologia e Medicina Tropical, São Paulo, São Paulo, Brazil; 2 Universidade de São Paulo, Faculdade de Medicina, Instituto de Medicina Tropical de São Paulo, Laboratório de Soroepidemiologia e Imunobiologia, São Paulo, São Paulo, Brazil; 3 Universidade de São Paulo, Faculdade de Medicina, Instituto de Medicina Tropical de São Paulo, Laboratório de Parasitologia, São Paulo, São Paulo, Brazil; 4 Universidade de São Paulo, Faculdade de Medicina, Hospital das Clínicas, Instituto Central, Serviço de Transplante Renal, São Paulo, São Paulo, Brazil

**Keywords:** Leishmaniasis, Kidney transplantation, Immunocompromised host, Serological tests, Polymerase chain reaction, Donor and recipient

## Abstract

This study examines the prevalence of anti-*Leishmania* IgG antibodies and *Leishmania* spp. infections among Brazilian kidney transplant recipients and their living donors before and after transplantation. A total of 48 donor-recipient pairs were recruited from July 14, 2022, to December 18, 2023. ELISA was used to test donors and recipients with a crude antigen of *Leishmania major*-like (*Lm*-ELISA), along with recombinant Lb6H (rLb6H-ELISA) and K39 (rK39-ELISA). Additionally, PCR was used to test recipients. Of the 48 donors, 25 (52.1%, 95%CI: 38.3–65.5) tested positive with *Lm*-ELISA, 4 (8.3%, 95%CI: 2.8–20.1) with rLb6H-ELISA, and 2 (4.2%, 95%CI: 0.4–14.8) with rK39-ELISA. Before transplantation, 31 recipients (64.6%, 95%CI: 50.4–76.6) were positive with *Lm*-ELISA, 5 (10.4%, 95%CI: 4.1–22.6) with rLb6H-ELISA, 1 (2.1%, 95%CI: <0.01–11.9) with rK39-ELISA, and 2 (4.2%, 95%CI: 0.4–14.8) with PCR. At 365 days post-transplant, 35 recipients underwent serological and molecular testing. Of these, 14 (40.0%, 95%CI: 25.5–56.5) tested positive with *Lm*-ELISA, 4 (11.4%, 95%CI: 3.9–26.5) with rLb6H-ELISA, 0 (0.0%, 95%CI: 0.0–11.8) with rK39-ELISA, and 2 (5.7%, 95%CI: 0.6–19.6) with PCR. Combining serological and molecular methods showed promising potential for early detection and ongoing monitoring of leishmaniasis in kidney transplant recipients and their donors. These findings highlight the urgent need for regulatory measures to implement *Leishmania*-specific donor screening and recipient monitoring using PCR and targeted serological tests, as well as close observation of signs and symptoms of leishmaniasis.

## INTRODUCTION

Leishmaniasis is a globally distributed vector-borne parasitic disease recognized as an important zoonosis. It presents a wide range of clinical manifestations, ranging from localized cutaneous involvement to severe damage of mucosal and visceral dissemination, the latter posing a life-threatening risk to affected individuals^1^. Classified by the World Health Organization (WHO) as a neglected tropical disease, leishmaniasis primarily affects the most socioeconomically vulnerable populations, making them a significant public health issue in endemic regions due to both their incidence and their potential to cause disability and lethality^2^.

The WHO estimates that approximately 50,000 to 90,000 new cases of visceral leishmaniasis (VL) and 600,000 to 1 million new cases of cutaneous leishmaniasis (CL) occur worldwide each year. However, these numbers are believed to be underreported, as only 11,922 VL and 272,098 CL cases were reported to the WHO in 2023^3^.

Due to the significant global incidence rates of leishmaniasis, attention to the infection has grown in recent decades. In addition to reports of recent epidemics in endemic areas, the disease has spread into previously non-endemic areas^2^. Moreover, individuals are more often afflicted by *Leishmania* spp. infections in conjunction with multiple comorbidities^4,5^. In line with these trends, although leishmaniasis is rarely reported among transplant recipients, VL cases among solid organ transplant patients have been rising worldwide. Between 1979 and 2006, the number of reported VL cases in this population increased approximately fourfold^6^.

In transplant patients, three mechanisms can lead to the development of leishmaniasis^6^. The first is a new *Leishmania* infection acquired by an immunocompromised recipient, either by being resident of an endemic country or after traveling to endemic regions. The second is the reactivation of a latent infection due to immunosuppressive therapy, which has been reported in liver, kidney, and stem-cell transplant recipients with prior exposure to *Leishmania*. The third is transmission via the transplanted organ or infected blood transfusions. In such cases, the donor is an asymptomatic carrier who can transmit the parasite to the recipient, potentially resulting in infection and parasitemia in the post-transplant period. Leishmaniasis may manifest in kidney recipients, particularly during the first year after transplantation, when immunosuppression is most pronounced^7^. Parasitemia may occur shortly after transplantation and is fundamental for the onset of overt disease, especially in VL cases^6^. In these individuals, the immunosuppressive therapy used to prevent organ rejection impairs T-lymphocyte function, hindering immune responses against intracellular microorganisms and thereby facilitating progression from infection to disease^7^.

The diagnosis of leishmaniasis depends on clinical manifestations and relies on clinical and epidemiological data, as well as laboratory tests with adequate sensitivity and specificity for confirmation^8^. However, in resource-limited countries, diagnostic confirmation is often not performed^1^. Early diagnosis is crucial for effective treatment^6^. Since outcomes and treatments can differ significantly depending on parasite species and potential toxicity issues, confirmation of clinical suspicion is essential. The best diagnostic approach combines parasitological and serological or molecular methods^1,9^.

This study employed serological and molecular methods to prospectively screen kidney recipients and their living donors at Hospital das Clinicas, Faculty of Medicine, University of Sao Paulo (HCFMUSP), for *Leishmania* infections and anti-*Leishmania* antibodies. Screening was conducted both before transplantation and 365 days after the procedure.

## MATERIALS AND METHODS

### Ethics

This study was approved by the Research Ethics Committee of the Faculty of Medicine, University of Sao Paulo, under approval Nº 4.750.576 (June 2, 2021) and amendment Nº 5.138.494 (December 1, 2021). Ethical approval was granted following Resolutions Nº 441/2011 and Nº 466/2012 of the Brazilian Ministry of Health and the National Health Council. All participants voluntarily provided written informed consent after reading and signing the form, authorizing the collection of blood samples and laboratory tests (Supplementary Files S1 and S2).

### Study design

This prospective study was conducted as part of the living kidney transplant program at HCFMUSP, where kidney transplant recipients and their living donors underwent medical monitoring, transplantation procedures, and laboratory tests. Enzyme-linked immunosorbent assays **(**ELISA) were performed at the Seroepidemiology and Immunobiology Laboratory, and polymerase chain reaction (PCR) was performed at the Parasitology Laboratory, both at Instituto de Medicina Tropical de Sao Paulo, Faculty of Medicine, University of Sao Paulo (IMT-FMUSP).

### Donors and recipients

Between July 14, 2022, and December 18, 2023, 48 adult living donors and their respective adult kidney transplant recipients (n = 96) were enrolled in this study. Both male and female participants were included, selected according to established technical criteria for organ donation following the completion of clinical evaluation and compatibility assessment with the respective recipient. Pre- and post-transplant blood samples were collected from July 19, 2022, to October 13, 2024. Individuals under 18 years of age, carriers of human immunodeficiency virus (HIV) infection, and deceased donors were excluded from the study.

Donors and their respective recipients were recruited as a convenience sample during scheduled transplant procedures. Data were obtained from medical records and included personal information (sex and age), demographic details (place of birth and residence, specifically rural or peri-urban locations), and relevant medical history, including prior treatment for CL, VL, or mucosal leishmaniasis (ML), year of sample collection, and date of transplantation. For recipients, clinical data were provided, including type of dialysis prior to transplantation, primary diagnosis, and relationship to the donor. Supplementary Tables S1 and S2 details the participant’s information.

Donor and recipient samples were relabeled using study codes to maintain participant anonymity and prevent bias.

### Enzyme-linked immunosorbent assay (ELISA)

ELISA with a *Leishmania major*-like antigen (*Lm*-ELISA) was performed using a crude alkaline extract of *L. major*-like (MHOM/BR/71/49) promastigotes^10^. The serum samples were twofold diluted, from 1:40 to 1:1,280. The cut-off for each plate was calculated as the mean absorbance of 10 negative standard sera at a 1:40 dilution plus two standard deviations. For each serum sample, the highest dilution that yielded an absorbance greater than the 1:40 cut-off was considered positive.

ELISA with recombinant antigens (rLb6H-ELISA and rK39-ELISA) was performed using rLb6H (*L. braziliensis*) and rK39 (*L. infantum-*syn. *chagasi*), both produced by the Infectious Disease Research Institute (IDRI), Seattle, USA. Serum samples were used at 1:100 dilution^11,12^. For each sample, the percentage absorbance of the positive standard and the reactivity index were calculated:


$$\text{ Absorbance }\%\text{ of the positive standard }=\frac{\text{ Sample absorbance }}{\text{ Positive control absorbance }}\times 100$$

$$\text{ Reactivity Index }(RI)=\frac{\text{ Absorbance \textbackslash{}\% of the positive standard }}{\text{ cut-off }}$$

Samples presenting RI ≥ 1 are considered positive. ELISA tests were performed on both donor and recipient serum samples.

### Polymerase chain reaction (PCR)

PCR was used to detect *Leishmania* DNA by targeting the conserved region of kinetoplast DNA (kDNA) minicircles. Peripheral blood samples were collected from recipients using two 3.5 mL tubes containing EDTA anticoagulant^13^. The collected blood and the extracted DNA were stored at −20 °C for future experiments. DNA was extracted using a commercial QIAamp DNA Mini Kit (Qiagen, Hilden, Germany) following the manufacturer’s instructions. The reaction employed kDNA 20 (5’ GGG KAG GGG CGT TCT SCG AA 3’) and kDNA 22 (5’ SSS WCT ATW TTA CAC CAA CC 3’) primers (Invitrogen, Thermo Fisher Scientific Corporation, Waltham, MA), producing a 120 bp fragment^13^. PCR was performed only on recipient samples.

### Chagas disease serological test

All donors and recipients were tested for Chagas disease using the LIAISON^®^ Automatic Immunoassay Analyzer, a chemiluminescence-based method. This assay employs a multi-epitope synthetic chimeric fusion protein, comprising antigens SAPA, TcF (TcLo1.2, TcE, TcD, and PEP-2), and IBMP (IBMP-8.1, IBMP-8.2, IBMP-8.3, and IBMP-8.4), all bound to magnetic particles. These antigens represent distinct immunodominant epitopes of *Trypanosoma cruzi*, improving the accuracy of antibody detection by encompassing a broad spectrum of immune responses. Index values ≥1 were considered reactive.

### Follow-up

Recipients who tested positive by ELISA with any antigen before transplantation, as well as those who tested negative but received an organ from a living donor who tested positive by ELISA with any antigen, were scheduled for follow-up sample collection at day 365 post-transplantation. Samples were tested by ELISA and PCR.

Recipients who tested positive by PCR before transplantation were scheduled for serial sample collection on days 30, 60, 90, 120, 180, and 365 post-transplantation for both ELISA and PCR testing. One recipient who was negative by PCR and serology but received a kidney from a donor with high ELISA titers for all three antigens was also scheduled for serial ELISA and PCR testing on the same time points.

### Statistical analysis

Fisher’s exact test, McNemar’s test, and 95% confidence intervals (95%CI) for proportions were performed using QuickCalcs (version 2025, GraphPad Software). R (version 4.4.1) and RStudio (version 2024.16.12) for Windows were used to generate Venn diagrams. A p-value < 0.05 was considered statistically significant.

## RESULTS

The demographic characteristics of the 96 participants (48 donor-recipient pairs) are presented in Table 1 and Supplementary Tables S1 and S2. Most donors and recipients were born and resided in the Southeast region of Brazil (Fisher’s exact test, p = 0.000). The diseases that led to renal failure and referred them for transplantation were hypertensive nephrosclerosis, glomerulonephritis, lupus nephritis, polycystic kidney disease, diabetic nephropathy, hydronephrosis, Alport’s syndrome, glomerulosclerosis, pyelonephritis, and vesicoureteral reflux (Supplementary Table S2). Of the 48 recipients, 41 (85.4%) were undergoing hemodialysis at the time of recruitment. The main immunomodulatory drugs the recipients used after transplantation were prednisone, tacrolimus, and mycophenolate mofetil.

**Table 1:** Demographic characteristics of donors (n = 48) and recipients (n = 48)

		Donors (n=48)	Recipients (n=48)
**Sex**	Female n (%)	28 (58.3%)	16 (33.3%)
	Male n (%)	20 (41.7%)	32 (66.7%)
	p-value*	0.1527	0.0020
**Mean age**		43	45
**min-max**		27-62	23-76
**Region of birth**	Southeast	33 (68.7%)	41 (85.4%)
	Northeast	12 (25.0%)	6 (12.5%)
	Central-West	1 (2.1%)	0
	South	1 (2.1%)	1 (2.1%)
	MI	1 (2.1%)	0
**Residence**	Southeast	41 (85.4%)	45 (93.7%)
	Northeast	1 (2.1%)	1 (2.1%)
	Central-West	2 (4.2%)	1 (2.1%)
	South	2 (4.2%)	0
	MI	2 (4.2%)	1 (2.1%)

n = number; MI = missing information; *Fisher’s exact test.

The median time for sample collection prior to transplantation was one day, with the 25^th^ percentile at 1.00 and the 75^th^ percentile at 2.75 days.

### Donors

**Figure 1:**
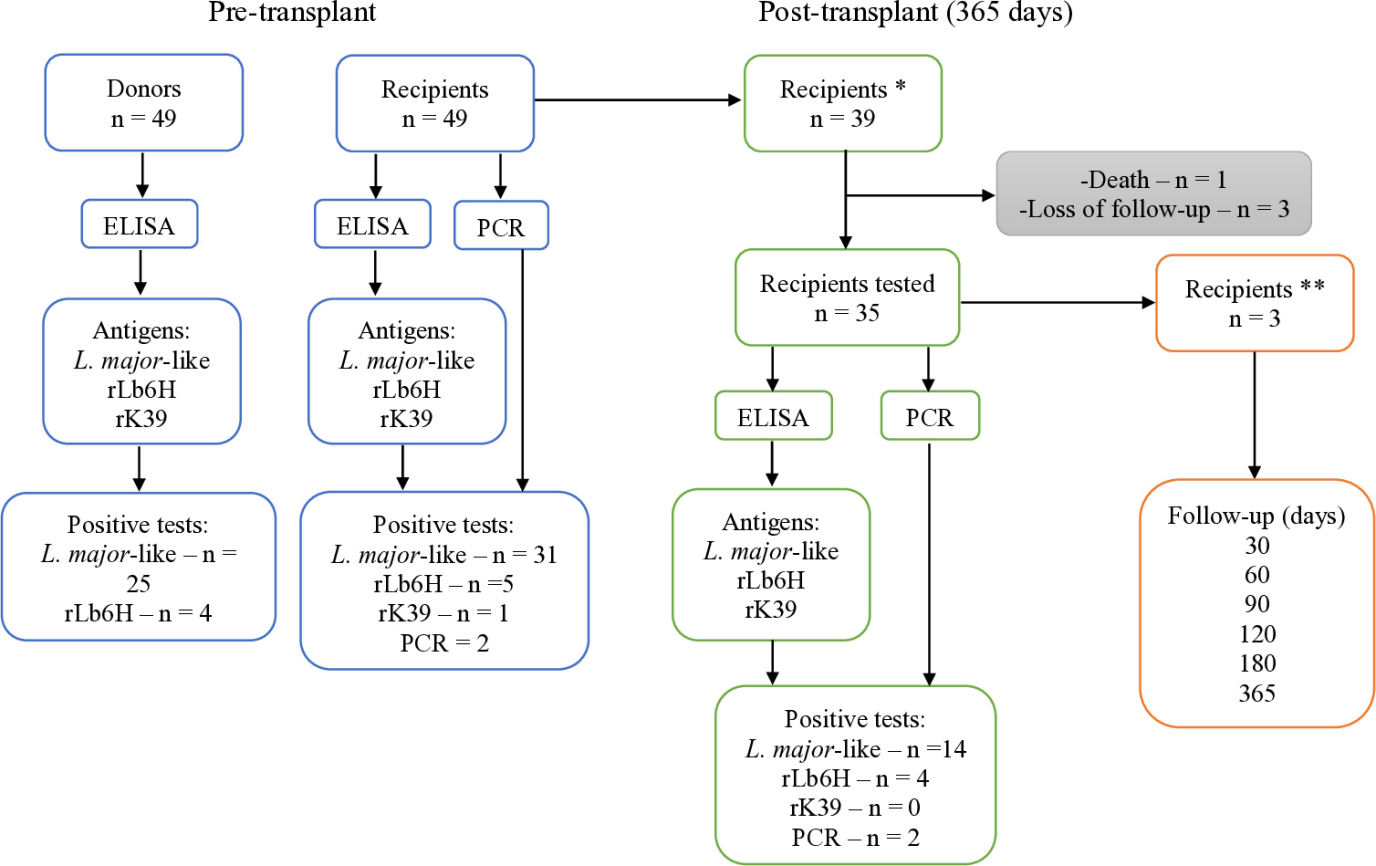
Flow diagram for reporting the evaluation of the prevalence of *Leishmania* spp. infection using ELISA and PCR before and 365 days post-transplant; n = number of samples; ELISA = enzyme-linked immunosorbent assay; PCR = polymerase chain reaction; *recipients who tested positive in any test or received an organ from a positive donor were tested 365 days post-transplant; **recipients who were PCR-positive before transplantation, and one PCR-negative recipient who received a kidney from a donor with high serological titers for all three ELISA antigens, were scheduled for sample collection on days 30, 60, 90, 120, 180, and 365 post-transplantation.

Among the 48 donors, pre-transplant positivity was 25 (52.1%, 95%CI: 38.3–65.5) by *Lm*-ELISA, 4 (8.3%, 95%CI: 2.8–20.1) by rLb6H-ELISA, and 2 (4.2%, 95%CI: 0.4–14.8) by rK39-ELISA. PCR testing was not performed on donor samples (Figure 1, Table 2). Three donors were positive in both *Lm*-ELISA and rLb6H-ELISA, and one was positive in all three antigens with high titers. Among the 48 donors, 46 (96%) reported not receiving treatment for CL, ML, or VL, and 28 (58%) reported having resided in rural or peri-urban areas (Supplementary Table S1). All donors tested negative for Chagas disease using the chemiluminescent immunoassay.

**Table 2:** ELISA and PCR results from kidney donors and their corresponding recipients at pre- and post-transplant periods

Donors						Recipients								
						Pre-transplant					Post-transplant (365 days)			
Patients	Lm-ELISA	rLb6H-ELISA	rK39-ELISA	PCR		Lm-ELISA	rLb6H-ELISA	rK39-ELISA	PCR		Lm-ELISA	rLb6H-ELISA	rK39-ELISA	PCR
1	**80**	**3,7**	N	ND		**40**	**1.1**	N	N		LF	LF	LF	LF
2	N	N	N	ND		**80**	**1.1**	N	**P**		N	**1.1**	N	N
3	N	N	N	ND		N	N	N	N		NC	NC	NC	NC
4	**80**	N	N	ND		**80**	N	N	N		**80**	N	N	N
5	**40**	N	N	ND		**40**	N	N	N		N	N	N	N
6	**40**	N	N	ND		**40**	N	N	N		LF	LF	LF	LF
7	**40**	N	N	ND		N	N	N	N		**40**	N	N	N
8	**40**	N	N	ND		**40**	N	N	N		**160**	N	N	N
9	**80**	N	N	ND		**80**	N	N	N		N	N	N	N
10	N	N	N	ND		N	N	N	N		NC	NC	NC	NC
11	**40**	N	N	ND		**40**	N	N	N		**40**	N	N	N
12	N	N	N	ND		**40**	N	N	N		**40**	N	N	N
13	N	N	N	ND		N	N	N	N		NC	NC	NC	NC
14	**40**	N	N	ND		**40**	**5.6**	N	N		N	**5.7**	N	N
15	N	N	N	ND		N	N	N	N		NC	NC	NC	NC
16	N	**3,7**	N	ND		**40**	N	N	N		**80**	N	N	N
17	**160**	N	N	ND		**40**	N	N	N		N	N	N	N
18	**40**	N	N	ND		N	N	N	N		N	N	N	N
19	N	N	N	ND		**40**	N	N	N		**320**	N	N	**P**
20	N	N	N	ND		**40**	N	N	**P**		**40**	N	N	N
21	**1280**	**13**	**13,2**	ND		N	N	N	N		**40**	N	N	N
22	**80**	N	N	ND		N	N	N	N		**320**	N	N	N
23	N	N	N	ND		**80**	**5.1**	N	N		**80**	**9.7**	N	N
24	N	N	N	ND		**40**	N	N	N		N	N	N	N
25	**160**	N	N	ND		**40**	N	N	N		N	N	N	N
26	**40**	N	N	ND		**40**	N	N	N		D	D	D	D
27	**80**	N	N	ND		**40**	N	N	N		N	N	N	N
28	N	N	N	ND		N	N	N	N		NC	NC	NC	NC
29	**80**	N	N	ND		**40**	N	N	N		N	N	N	N
30	**40**	N	N	ND		N	N	N	N		N	N	N	N
31	N	N	N	ND		N	N	N	N		NC	NC	NC	NC
32	**80**	N	N	ND		**40**	N	N	N		N	N	N	N
33	**40**	**1,5**	N	ND		**40**	N	N	N		N	N	N	N
34	N	N	N	ND		**160**	N	N	N		LF	LF	LF	LF
35	N	N	N	ND		**40**	N	N	N		N	N	N	N
36	N	N	N	ND		**40**	N	N	N		N	N	N	N
37	**80**	N	N	ND		N	N	N	N		N	N	N	N
38	N	N	N	ND		N	N	N	N		NC	NC	NC	NC
39	**40**	N	N	ND		**40**	**1.0**	N	N		N	**2.7**	N	N
40	**40**	N	N	ND		**80**	N	N	N		N	N	N	N
41	N	N	N	ND		**40**	N	N	N		**80**	N	N	N
42	N	N	N	ND		**640**	N	N	N		N	N	N	**P**
43	**80**	N	N	ND		N	N	N	N		N	N	N	N
44	N	N	N	ND		N	N	N	N		NC	NC	NC	NC
45	N	N	N	ND		N	N	N	N		NC	NC	NC	NC
46	N	N	**1,2**	ND		N	N	N	N		**40**	N	N	N
47	**40**	N	N	ND		**80**	N	N	N		N	N	N	N
48	N	N	N	ND		**160**	N	**1.1**	N		**160**	N	N	N
P/T	25/48	4/48	2/48			31/48	5/48	1/48	2/48		14/35	4/35	0/35	2/35

Results are presented as titers for L. major-like ELISA, as reactivity indexes for rLb6H- ELISA and -rK39 ELISA; P = positive; N = negative results; ND = not done; LF = loss of follow-up; NS = not summoned; D = death; P/T = number of positive samples/total number of samples.

### Recipients in the pre-transplant period (N = 48)

Among the 48 recipients evaluated in the pre-transplant period, positivity was 31 (64.6%, 95%CI: 50.4–76.6) by *Lm*-ELISA, 5 (10.4%, 95%CI: 4.1–22.6) by rLb6H-ELISA, and 1 (2.1%, 95%CI: < 0.01–11.9) by ELISA-rK39 (Figure 1, Table 2, Supplementary Table S2). No statistical difference was observed between donors and recipients for any of the three antigens (McNemar’s test: *Lm*-ELISA, p = 0.2636; rLb6H-ELISA, p = 0.3711; and rK39-ELISA, p = 1.000). PCR positivity in the pre-transplant period was 2 (4.2%, 95%CI: 0.4–14.8). Five recipients were positive by both *Lm*-ELISA and rLb6H-ELISA, and one was positive by both *Lm*-ELISA and rK39-ELISA. Two recipients were positive by both PCR and *Lm*-ELISA, one of which was also positive for rLb6H-ELISA.

Among the 48 recipients, 47 (98%) reported not receiving treatment for CL, ML, or VL, and 27 (56%) reported having resided in rural or peri-urban areas (Supplementary Table S2). All recipients tested negative for Chagas disease using the chemiluminescent immunoassay.

### Recipients in the post-transplant period (N = 35)

**Figure 2:**
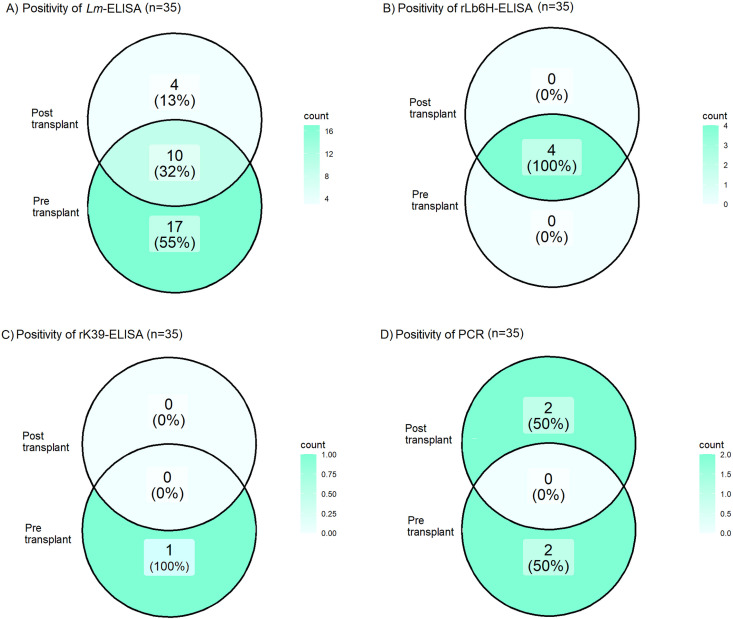
Venn diagram showing pre- and post-transplant agreement among recipients for *Lm*-ELISA, rLb6H-ELISA, rK39-ELISA, and PCR. ELISA = enzyme-linked immunosorbent assay; PCR = polymerase chain reaction; n = number of samples.

Of the 39 recipients scheduled for collecting samples for ELISA and PCR tests 365 days after transplantation, 35 attended the follow-up (Figure 1). Of these, the positivity rate in *Lm*-ELISA was 14 (40.0%, 95%CI: 25.5–56.5). When comparing pre- and post-transplantation results for these 35 individuals using *Lm*-ELISA, 27 (77.4%, 95% CI: 60.7–88.2) were positive pre-transplantation, significantly higher than post-transplant rates (McNemar’s test, p = 0.0088). Among the 27 recipients positive in *Lm*-ELISA pre-transplant, 10 remained positive post-transplant, six maintained identical titers, four exhibited increased titers, and 17 seroconverted to negative (Figure 2). Four recipients initially negative in *Lm*-ELISA seroconverted to positive 365 days after transplantation, three of whom had received kidneys from *Lm*-ELISA-positive donors.

For rLb6H-ELISA, positivity was 4/35 (11.4%, 95%CI: 3.9–26.5). All had tested positive pre-transplant (Figure 2); two maintained similar reactivity indices (RI), while two showed increased RI 365 post-transplant. The three recipients who were negative for rLb6H-ELISA before transplantation and received kidneys from rLb6H-ELISA-positive donors remained negative 365 days post-transplant.

For rK39-ELISA, no recipient (0.0%, 95%CI: 0.0–11.8) tested positive 365 days post-transplantation (Figure 2). The two recipients who were negative pre-transplant and received kidneys from rK39-ELISA-positive donors remained negative 365 post-transplant.

**Figure 3:**
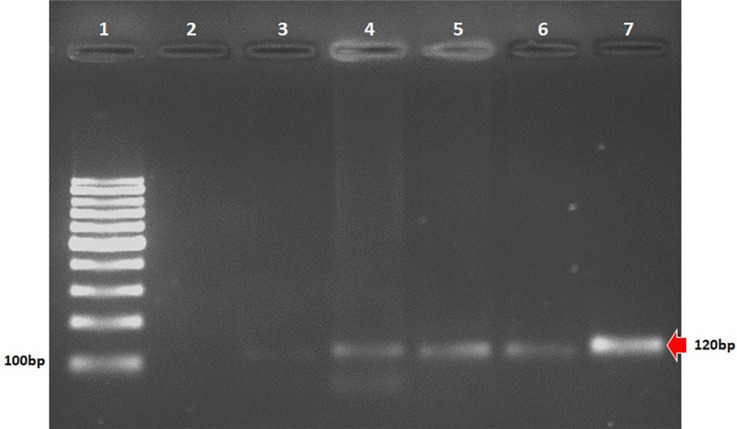
Amplification products from peripheral blood from kidney recipients; (1) Molecular weight; (2) Negative control; (3–4) Pre-transplant recipient samples (patients 2 and 20); (5–6) Post-transplant recipient samples (patients 19 and 42); (7) Positive control; Bp = base pairs.

PCR positivity at day 365 was 2/35 (5.7%, 95%CI: 0.6–19.6); however, these were not the same individuals who tested PCR-positive before transplantation (McNemar’s test, p = 0.6171) (Figure 3).

Two recipients who were PCR-positive pre-transplant and one PCR-negative recipient who received a kidney from a donor with high serological titers were followed up for 30, 60, 90, 120, 180, and 365 days. The two recipients who were PCR-positive pre-transplant became negative throughout follow-up. For rLb6H-ELISA, one recipient remained positive with stable titers, while the other remained negative throughout collections. In the *Lm*-ELISA, both recipients presented with low or negative titers during follow-up. The initially negative recipient who received a kidney from a donor with high serological titers remained negative in PCR, rLb6H-ELISA, and rK39-ELISA, and presented low and negative *Lm*-ELISA titers during follow-up (Table 3).

**Table 3:** ELISA and PCR results from kidney donors and their corresponding recipients at pre-transplant and follow-up from six post-transplant collections

ID	Donors				Recipients																											
					Pre-transplant period				Post-transplant period follow-up (days)																							
									30				60				90				120				180				365			
	*Lm*	rLb6H	rK39	PCR	*Lm*	rLb6H	rK39	PCR	*Lm*	rLb6H	rK39	PCR	*Lm*	rLb6H	rK39	PCR	*Lm*	rLb6H	rK39	PCR	*Lm*	rLb6H	rK39	PCR	*Lm*	rLb6H	rK39	PCR	*Lm*	rLb6H	rK39	PCR
2	N	N	N	ND	**80**	**1.1**	N	**P**	**80**	**1,2**	N	N	80	**1.2**	N	N	40	**1,3**	N	N	N	**1,4**	N	N	40	**1.5**	N	N	N	**1,1**	N	N
20	N	N	N	ND	**40**	N	N	**P**	N	N	N	N	40	N	N	N	80	N	N	N	80	N	N	N	80	N	N	N	40	N	N	N
21	**1280**	**13.0**	**13.2**	**ND**	N	N	N	N	**40**	N	N	N	N	N	N	N	N	N	N	N	40	N	N	N	N	N	N	N	40	N	N	N

Results are presented as titers for L. major-like ELISA, as reactivity indexes for rLb6H- ELISA and -rK39 ELISA; P = positive; N = negative results; ND = not done.

All recipients were examined by both an infectious disease specialist and an otorhinolaryngologist for skin or mucosal lesions indicative of CL or ML. If VL was suspected, a clinical examination of the liver and spleen was conducted, along with blood tests for anemia, leukopenia, and thrombocytopenia. No recipients exhibited clinical signs, symptoms, or laboratory findings suggesting leishmaniasis during clinical evaluations.

## DISCUSSION

Leishmaniasis is rarely reported in solid organ transplant recipients^6^. According to Clemente *et al*.^14^, no confirmed donor-derived infections have been documented. Nevertheless, studies have reported reactivation with clinical manifestations, primarily of VL, in kidney transplant patients, often associated with high post-diagnosis mortality^15-18^. CL is rare among organ transplant recipients^6,19^. Most post-transplant leishmaniasis cases after kidney transplants reported in the literature come from Brazil, Spain, France, Italy, and Tunisia, all of which are endemic for the disease^2,20^.

As of 2022, Brazil ranked fourth in the absolute number of kidney transplants among 35 countries, following the United States, China, and India, with 6,047 procedures performed^21^. During the study recruitment period, from July 2022 to December 2023, 48 kidney transplant candidates were selected. In the same period, Brazil performed 8,983 kidney transplants, 1,253 of which involved living donors^21^. In Sao Paulo State, 2,971 transplants were performed, including 546 from living donors^21^. Therefore, this study included approximately 4% of all candidates for inter-vivo kidney transplants in Brazil and around 9% in Sao Paulo State. As the number of solid organ transplants continues to rise, the potential risk of the latent infection reactivation and the emergence of overt disease could become an increasing concern post-transplant.

Immunosuppressive drugs used in transplant recipients—such as cyclosporine, azathioprine, corticosteroids, and tacrolimus—impair immune defense by inhibiting T-cell activation and proliferation, thus compromising the host’s response against intracellular pathogens. Cyclosporine and tacrolimus share a similar action mechanism, as both inhibit calcineurin and block the production of interleukin-2 (IL-2), a cytokine essential for T-lymphocyte activation and proliferation^6^.

Routine screening for leishmaniasis in kidney transplant donors is not currently recommended^14^. Given the limited data on donor-transmitted infections and the high prevalence of asymptomatic infected donors, a positive serology or other markers of previous exposure without evidence of active infection do not contraindicate donation^14^. However, living donors with active leishmaniasis should undergo treatment before donation, and recipients of donors with previous VL should be clinically monitored for infection, particularly during the first post-transplant year^14^.

Previous studies have shown variations in the prevalence of anti-*Leishmania* antibodies. In Minas Gerais State, an endemic region for VL, one study found a 0% prevalence among deceased liver transplant donors and 2% among recipients using indirect immunofluorescence (IFAT)^14^. Similarly, another study in the same region reported a 2.1% prevalence of asymptomatic *L. infantum* infection among blood donors, as determined by IFAT^22^.

Studies from Spain and Brazil have revealed notable rates of anti-*Leishmania* antibody positivity among patients with chronic kidney disease, many of whom are transplant candidates. Reported rates ranged from 4.8% in Spain to 32% in Brazil, highlighting the risk of asymptomatic *Leishmania* infection in this population^23,24^. In Italy, a serological survey using Western blot detected a 14.3% prevalence of asymptomatic *L. infantum* infection in chronic kidney patients, 60% of whom were transplant candidates^25^. A systematic review also reported high titers (median 400) in IFAT-based serological tests for visceral leishmaniasis among organ transplant recipients, with 92% (45/49) of patients testing positive^6^.

In this study, we employed ELISA based on *L. major*-like antigen. However, its reported sensitivity has varied, ranging from 66.9% for CL^26^, 78.7% for tegumentary forms (CL and ML)^27^, and 93.3% for ML^28^. In regions endemic for *L. braziliensis*, the use of homologous antigens may improve diagnostic performance compared to *L. major*-like antigens^27^. Therefore, we recommend complementing *Lm*-ELISA with assays based on specific recombinant proteins to improve both sensitivity and specificity in the diagnostic process.

This study included diagnostic tests targeting antibodies against parasites responsible for cutaneous disease—which is more prevalent and occurs in all Brazilian states^29^—and visceral disease, which is geographically restricted^2^. As no single test is considered the gold standard for detecting asymptomatic or latently infected individuals^30,31^, combining multiple methods is recommended for diagnosis^14^.

It is important to note that ELISA with crude antigens may yield cross-reactions with other infections, including malaria, tuberculosis, paracoccidioidomycosis, histoplasmosis, and, most notably, Chagas disease^32^. Although *Lm*-ELISA has shown high sensitivity in symptomatic patients (90.4%), particularly for VL and ML, its specificity (56.0%) may be compromised by cross-reactions^33^. In this study, all donors and recipients tested negative for Chagas disease.

During the pre-transplant period, *Lm*-ELISA was positive in 52.1% of donors and 64.6% of recipients. Among the 25 positive donors, 60.0% were born in or had resided in areas with transmission of tegumentary leishmaniasis (TL) and/or VL, and 20.0% were from areas with canine visceral leishmaniasis (CVL). Similarly, of the 31 positive recipients, 45.2% were born or resided in TL and/or VL transmission areas, and 16.0% in CVL transmission areas. Among the 14 post-transplant positive recipients, 49.9% were born or had resided in TL and/or VL transmission areas, and 14.3% in CVL transmission areas. These findings underscore a potential risk for the transmission or reactivation of leishmaniasis^19^.

The rLb6H antigen, although not yet studied in kidney transplant populations, shows high diagnostic accuracy for CL and ML in Brazil, with sensitivity ranging from 98.6% to 100% and specificity from 93.9% to 100%^10,32^. Conversely, the rK39 antigen effectively identifies symptomatic VL but has lower sensitivity in asymptomatic cases^34^. It has shown a sensitivity of 94.3% and a specificity of 95.2% among patients with clinical manifestations of VL and healthy controls, respectively^12^. Other Brazilian studies^24,35^ have reported no rK39 positives using the rapid test, which is less sensitive than ELISA.

During the pre-transplant period, serological results for recombinant antigens showed 8.3% positivity among donors and 10.4% among recipients for rLb6H, while positivity rates for rK39 were 4.2% among donors and 2.1% among recipients. These findings align with the epidemiology of leishmaniasis in Brazil, which indicates a higher CL prevalence compared to VL^29^, as well as a higher number of donors and recipients recruited from the Southeast region. Notably, the four donors positive for rLb6H-ELISA and one of the two positive for rK39-ELISA were born or resided in TL transmission areas. The other rK39-ELISA-positive donor was born in a CVL transmission area.

For rLb6H-ELISA, four recipients were positive both before and after transplantation. However, none of the four recipients who were initially negative in rLb6H-ELISA- or donors who were positive in rK39-ELISA showed seroconversion after one year. Among the four recipients positive in both *Lm*-ELISA and rLb6H-ELISA prior to transplantation, only the recipient who showed a significant increase in antibody levels detected by rLb6H-ELISA maintained the titer in *Lm*-ELISA. The other three remained positive only with rLb6H-ELISA, indicating that the immune response to recombinant antigens may persist despite immunosuppressive conditions. These antigens, cloned from *Leishmania* genes, offer improved assay accuracy and reproducibility^36^.

In this study, PCR testing in recipients revealed a prevalence of 4.2% of asymptomatic infections during the pre-transplant period. Other studies found varying rates: 23.5% in deceased donors and 6% in recipients in a Brazilian liver transplant study^14^, 1.8% in blood donors from an endemic region^22^, and around 2.5%^25^ and 3.3%^31^ in two Italian studies using PCR. The latter studies also employed a whole blood assay (WBA), which has been validated to assess anti-leishmanial cell-mediated immunity by measuring cytokine and chemokine levels in plasma from blood cells stimulated with parasitic antigens^31^. Asymptomatic individuals in *Leishmania*-endemic areas may experience transient parasitemia^37^. Residual parasites can persist due to variations in parasite load, host characteristics, and factors such as age, nutritional status, sex, comorbidities, pregnancy, coinfections, and multifactorial influences^38^. Immunosuppressed patients are at higher risk of reactivation due to weakened immune responses^39^. PCR assays that amplify the conserved region of kinetoplast DNA minicircles (approximately 10,000 copies per cell) are highly sensitive for detecting *Leishmania* ^13^. Both recipients who were PCR-positive before transplantation and one recipient who tested PCR-positive after transplantation were born and resided in TL transmission areas.

In the pre-transplant period, two recipients tested positive for PCR, and both showed low antibody titers on *Lm*-ELISA. These recipients were negative in all six follow-up collections (from 30 to 365 days). In the post-transplant period, two other recipients also tested positive for PCR: one had a higher antibody titer on *Lm*-ELISA, while the other tested negative. These results demonstrate that the relationship between parasitemia and antibody production is highly complex in the context of immunosuppression and may vary depending on the host’s immune response and the stage of the disease. None of the four PCR-positive recipients presented any symptoms.

Notably, 120 days after transplantation, a recipient who was positive in *Lm*-ELISA and rLb6H-ELISA returned for consultation and tested positive with PCR, with an increase in *Lm*-ELISA (80 to 160) and rLb6H-ELISA (5.1 to 11.2) titers. By day 180 after transplantation, PCR was negative, the *Lm*-ELISA titer remained stable, and the Lb6H-ELISA titer decreased to 9.1. No symptoms or signs of disease were observed.

Leishmaniasis in kidney transplant recipients can develop anywhere from a few days to 246 months after transplantation^20^, but it usually occurs as a late complication, with a median onset of 19 months^6^. In our study, no recipients or positive donors exhibited clinical manifestations of leishmaniasis after the one-year follow-up. However, three of the seven negative recipients who received organs from *Lm*-ELISA-positive donors seroconverted by day 365 post-transplant. One of these recipients was monitored for one year with six follow-up collections due to high serological titers, yet no symptoms or clinical signs of VL were observed.

Limitations of this study include a small sample size, a relatively short follow-up period, and a high rate of loss to follow-up. Extending the follow-up duration may enhance early detection and mitigation of leishmaniasis-related risks.

Discordant results between molecular and immunological methods for identifying asymptomatic *Leishmania* infections have been reported^31^. Such discordance may occur when a more specific method is compared with a less specific one^14^. Consequently, relying on a single serological test may not accurately reflect the true prevalence of infection in at-risk populations, potentially introducing misclassification bias in epidemiological studies^40^. Therefore, the detection of asymptomatic *Leishmania* infection may vary between studies due to differences in detection methods, antigens, and molecular targets^30^.

## CONCLUSION

This study employed serological and molecular methods to prospectively screen kidney recipients and their living donors for *Leishmania* infections and anti-*Leishmania* antibodies before transplantation and 365 days after.

The methods herein employed offer preliminary insights into the potential for early detection and monitoring of leishmaniasis in kidney donors and recipients. However, further research with larger sample sizes and extended follow-up periods is necessary to confirm these findings. ELISA-Lb6H showed a good performance, consistent with the higher prevalence of CL compared to VL. The results suggest a potential benefit in implementing regulations for donor screening and recipient monitoring using PCR and specific serological tests, alongside careful observation of leishmaniasis signs and symptoms. However, further studies are needed to establish the efficacy and cost-effectiveness of such measures before widespread implementation.

## Data Availability

The complete anonymized dataset supporting the findings of this study is available at https://doi.org/10.48331/SCIELODATA.MN0GSG
