# Supplementary material for: Screening for anti-Leishmania antibodies and Leishmania infections in kidney transplant recipients and donors from Brazil
Source: Rev Inst Med Trop Sao Paulo. 2025 Oct 3;67:e67. doi: 10.1590/S1678-9946202567067 (PMC12499513; doi:10.1590/S1678-9946202567067)
Supplement: Supplementary file 1 [file 1678-9946-rimtsp-67-S1678-9946202567067-suppl01.pdf]

REVISTA DO INSTITUTO DE MEDICINA TROPICAL DE SÃO PAULO

JOURNAL OF THE SÃO PAULO INSTITUTE OF TROPICAL MEDICINE

SUPPLEMENTARY MATERIAL

https://doi.org/10.48331/SCIELODATA.MN0GSG

Screening for anti-*Leishmania* antibodies and *Leishmania* infections in kidney transplant recipients and donors from Brazil

Gustavo Henrique Johanson [1], Maria Carmen Arroyo Sanchez [2], Regina Maia de Souza [3], Beatriz Julieta Celeste [2], Ruth Tamara Valencia-Portillo [2], Elias David-Neto [4], Ligia Camera Pierrotti [1,4], Valdir Sabbaga Amato [1,3]

[1]Universidade de São Paulo, Faculdade de Medicina, Instituto de Medicina Tropical de São Paulo, Departamento de Infectologia e Medicina Tropical, São Paulo, São Paulo, Brazil

[2]Universidade de São Paulo, Faculdade de Medicina, Instituto de Medicina Tropical de São Paulo, Laboratório de Soroepidemiologia e Imunobiologia, São Paulo, São Paulo, Brazil

[3]Universidade de São Paulo, Faculdade de Medicina, Instituto de Medicina Tropical de São Paulo, Laboratório de Parasitologia, São Paulo, São Paulo, Brazil

[4]Universidade de São Paulo, Faculdade de Medicina, Hospital das Clínicas, Instituto Central, Serviço de Transplante Renal, São Paulo, São Paulo, Brazil

Correspondence to: Maria Carmen Arroyo Sanchez
Universidade de São Paulo, Faculdade de Medicina, Instituto de Medicina Tropical de São Paulo, Laboratório de Soroepidemiologia e Imunobiologia, Av. Dr. Enéas Carvalho de Aguiar, 470, CEP 05403-000, São Paulo, SP, Brazil

E-mail: arroyo@usp.br

Received: 5 June 2025

Accepted: 19 August 2025

Supplementary Table S1 - Raw data obtained by baseline information and test results applied to donors' samples studied.

	Baseline information											Test results			
ID	Gender	Age	Collection year	Trans-plantation year	Place of birth	Residence	Lived in rural area	Lived in peri-urban area	Treatment CL	Treatment ML	Treatment VL	*Lm*-ELISA	rLb6H-ELISA	rK39-ELISA	PCR
1	F	33	2022	2022	Caraguatatuba - SP	Sao Sebastiao - SP	No	No	No	No	No	**80**	**3.7**	0.2	ND
2	M	51	2022	2022	Sao Paulo - SP	Itapevi - SP	No	Yes	No	No	No	N	0.4	0.8	ND
3	M	48	2022	2022	Natal - RN	Franco da Rocha - SP	No	No	No	No	No	N	0.3	0.3	ND
4	M	40	2022	2022	Sao Paulo - SP	Sao Paulo - SP	No	Yes	No	No	No	**80**	0.2	0.0	ND
5	F	49	2022	2022	Penedo - AL	Diadema - SP	No	Yes	No	No	No	**40**	0.3	0.0	ND
6	F	50	2022	2022	VItória da Conquista - BA	Taboao da Serra - SP	No	Yes	No	No	No	**40**	0.3	0.1	ND
7	M	54	2022	2022	Sao Domingos do Prata - MG	Almirante Tamandare - PR	Yes	No	No	No	No	**40**	0.8	0.1	ND
8	M	40	2022	2022	Paranavai - PR	Sao Paulo - SP	Yes	No	No	No	No	**40**	0.6	0.1	ND
9	M	38	2022	2022	Guaruja - SP	Santos - SP	No	No	No	No	No	**80**	0.4	0.0	ND
10	F	39	2022	2022	Giraldo Ponciano - AL	Campo Grande - AL	No	No	No	No	No	N	0.7	0.1	ND
11	F	43	2022	2022	Barueri -SP	Barueri -SP	No	Yes	No	No	No	**40**	0.1	0.2	ND
12	F	36	2022	2022	Penapolis - SP	Penapolis - SP	No	No	No	No	No	N	0.4	0.2	ND
13	F	33	2022	2022	Guarulhos - SP	Guarulhos - SP	No	No	No	No	No	N	0.4	0.0	ND
14	F	41	2022	2022	Sao Felipe - BA	Francisco Morato - SP	Yes	Yes	No	No	No	**40**	0.6	0.0	ND
15	M	55	2022	2022	Sao Paulo - SP	Sao Paulo - SP	No	Yes	No	No	No	N	0.6	0.0	ND
16	F	38	2022	2023	Sao Paulo - SP	Araraquara - SP	No	No	No	No	No	N	**3.7**	0.2	ND
17	F	38	2022	2022	Guarulhos - SP	Guarulhos - SP	No	Yes	No	No	No	**160**	0.5	0.1	ND
18	F	38	2022	2022	Uniao dos Palmares - AL	Sao Paulo - SP	Yes	No	No	No	No	**40**	0.5	0.1	ND
19	F	34	2023	2023	Araraquara - SP	Araraquara - SP	Yes	No	No	No	No	N	0.2	0.1	ND
20	M	39	2023	2023	Sao Paulo - SP	Sao Paulo - SP	No	Yes	No	No	No	N	0.4	0.1	ND
21	F	36	2023	2023	Natividade da Serra - SP	Sao Paulo - SP	Yes	No	No	No	No	**1280**	**13.0**	**13.2**	ND
22	M	61	2023	2023	Ibitinga - SP	Ibitinga - SP	Yes	No	No	No	No	**80**	0.1	0.1	ND
23	M	48	2023	2023	Remanso - BA	Carapicuiba - SP	Yes	Yes	No	No	No	N	0.4	0.1	ND
24	F	56	2023	2023	Pedra Branca - PB	Sao Paulo - SP	Yes	No	No	No	No	N	0.3	0.1	ND
25	F	33	2023	2023	Arco Verde - PE	Caraguatatuba - SP	Yes	Yes	No	No	No	**160**	0.2	0.0	ND
26	F	48	2023	2023	Senador Firmino - MG	Senador Firmino - MG	Yes	Yes	No	No	No	**40**	0.3	0.4	ND
27	F	47	2023	2023	Sao Paulo - SP	Sao Paulo - SP	No	No	No	No	No	**80**	0.7	0.0	ND
28	M	37	2023	2023	Santa Branca - SP	Jacarei -SP	No	No	No	No	No	N	0.3	0.0	ND
29	M	34	2023	2023	Sao Bernardo do Campo - SP	Sao Bernardo do Campo - SP	No	No	No	No	No	**80**	0.4	0.0	ND
30	F	50	2023	2023	Presidente Epitacio - SP	Pirapozinho - SP	Yes	No	No	No	No	**40**	0.6	0.1	ND
31	M	38	2023	2023	Rio de Janeiro - RJ	Marialva - PR	No	No	No	No	No	N	0.3	0.1	ND
32	F	45	2023	2023	Juazeiro - BA	Sao Paulo - SP	No	Yes	No	No	No	**80**	0.4	0.0	ND
33	F	56	2023	2023	Maceio - AL	Sao Paulo - SP	No	No	No	No	No	**40**	**1.5**	0.2	ND
34	M	41	2023	2023	Queimadas - BA	Taboao da Serra - SP	No	No	No	No	No	N	0.7	0.5	ND
35	F	33	2023	2023	Sao Paulo - SP	Sao Paulo - SP	No	No	No	No	No	N	0.1	0.1	ND
36	M	50	2023	2023	Machacalis - MG	Serra - ES	Yes	Yes	No	No	No	N	0.2	0.2	ND
37	M	42	2023	2023	Penapolis - SP	Penapolis - SP	Yes	MI	No	No	No	**80**	0.7	0.5	ND
38	F	48	2023	2023	Sete Lagoas - MG	Mogi das Cruzes - SP	No	No	No	No	No	N	0.2	0.2	ND
39	F	27	2023	2023	Sao Paulo - SP	MI	MI	MI	MI	MI	MI	**40**	0.5	0.1	ND
40	M	40	2023	2023	Ubatuba - SP	Caraguatatuba -SP	No	No	No	No	No	**40**	0.7	0.0	ND
41	M	47	2023	2023	MI	MI	MI	MI	MI	MI	MI	N	0.6	0.1	ND
42	F	40	2023	2023	Caçapava - SP	Caçapava - SP	No	Yes	No	No	No	N	0.7	0.0	ND
43	F	33	2023	2023	Sao Paulo -SP	Sao Paulo -SP	No	Yes	No	No	No	**80**	0.6	0.2	ND
44	F	35	2023	2023	Itajuba - MG	Guaratingueta -SP	Yes	No	No	No	No	N	0.2	0.2	ND
45	F	47	2023	2023	Cuiaba - MT	Jucimeira - MT	Yes	No	No	No	No	N	0.2	0.1	ND
46	M	31	2023	2023	Aruja - SP	MI	MI	MI	No	No	No	N	0.1	**1.2**	ND
47	M	54	2023	2024	Osasco- SP	Osasco - SP	No	No	No	No	No	**40**	0.1	0.0	ND
48	F	62	2023	2023	Ibitinga - SP	Tremembe - SP	Yes	Yes	No	No	No	N	0.1	0.0	ND

ID = identification number; M = male; F = female; age = in years; CL = cutaneous leishmaniasis; ML = mucosal leishmaniasis; VL = visceral leishmaniasis; N = negative; ND = not done; Lm-ELISA = ≥ 40 positive; rLb6H-ELISA = ≥ 1 positive; rK39-ELISA = ≥ 1 positive; SP = Sao Paulo State; PR = Parana State; AL = Alagoas State; MG = Minas Gerais State; ES = Espirito Santo State; MT = Mato Grosso State; BA = Bahia State; RJ = Rio de Janeiro State; PB = Paraiba State; RN = Rio Grande do Norte State, MI = missing information.

This is an open-access article distributed under the terms of the Creative Commons Attribution License.

Supplementary Table S2 - Raw data obtained by baseline information and test results applied to recipient's samples studied.

	Baseline information														Test results - pre-transplant				Test results - post-transplant			
ID	Gender	Age	Collection year	Transplantation year	Place of birth	Residence	Lived in rural area	Lived in peri-urban area	Treatment CL	Treatment ML	Treatment VL	Type of dialysis	Clinical diagnosis	Donator Relationship	*Lm*-ELISA	rLb6H-ELISA	rK39-ELISA	PCR	*Lm*-ELISA	rLb6H-ELISA	rK39-ELISA	PCR
1	M	59	2022	2022	Sao Paulo - SP	Sao Sebastiao - SP	No	No	No	No	No	HD	Hypertensive nephrosclerosis	daughter	**40**	**1.1**	0.1	N	ND	ND	ND	ND
2	F	53	2022	2022	Sao Paulo - SP	Itapevi - SP	No	Yes	No	No	No	CAPD	Diabetes mellitus type I	brother	**80**	**1.1**	0.1	**P**	N	**1.1**	0.3	N
3	M	76	2022	2022	Itarema - CE	Franco da Rocha - SP	Yes	No	No	No	No	No	Autosomal dominant polycystic kidney disease	friend	N	0.9	0.1	N	ND	ND	ND	ND
4	M	68	2022	2022	Belem de Maria - PE	Sao Paulo - SP	Yes	Yes	No	No	No	HD	Hydronephrosis	son	**80**	0.3	0.2	N	**80**	0.2	0.4	N
5	M	25	2022	2022	Diadema -SP	Diadema - SP	No	Yes	No	No	No	HD	Vesicle uretral reflux	uncle	**40**	0.4	0.0	N	N	0.2	0.1	N
6	M	33	2022	2022	Taboao da Serra - SP	Embu das Artes - SP	No	No	No	No	No	HD	Chronic glomerulonephritis	mother	**40**	0.8	0.1	N	ND	ND	ND	ND
7	F	59	2022	2022	Sao Domingos do Prata - MG	Embu das Artes - SP	Yes	Yes	No	No	No	HD	Chronic interstitial nephritis	brother	N	0.3	0.1	N	**40**	0.4	0.1	N
8	M	23	2022	2022	Sao Paulo - SP	Sao Paulo - SP	No	No	No	No	No	HD	Alport syndrome	father	**40**	0.3	0.1	N	**160**	0.5	0.36	N
9	F	38	2022	2022	Guaruja - SP	Guaruja - SP	No	No	No	No	No	HD	Uncleared chronic renal insufficiency	brother	**80**	0.1	0.2	N	N	0.1	0.4	N
10	M	40	2022	2022	Campo Grande - AL	Campo Grande - AL	Yes	No	No	No	No	No	Lupus nephritis	husband	N	0.4	0.0	N	ND	ND	ND	ND
11	F	42	2022	2022	Jandira - SP	Barueri - SP	No	Yes	No	No	No	HD	Lupus nephritis	sister	**40**	0.7	0.4	N	**40**	0.2	0.1	N
12	F	29	2022	2022	Penapolis - SP	Penapolis - SP	No	No	No	No	No	HD	Chronic pyelonephritis	MI	**40**	0.3	0.1	N	**40**	0.8	0.2	N
13	F	70	2022	2022	Maceio - AL	Guarulhos - SP	No	No	No	No	No	HD	Hypertensive nephrosclerosis	daughter	N	0.8	0.2	N	ND	ND	ND	ND
14	F	25	2022	2022	Sao Paulo - SP	Morro Doce - SP	No	Yes	No	No	No	HD	Lupus nephritis	mother	**40**	**5.6**	0.2	N	N	**5.7**	0.3	N
15	M	50	2022	2022	Sao Paulo - SP	Sao Paulo - SP	No	Yes	No	No	No	HD	Hypertensive nephrosclerosis	brother	N	0.3	0.5	N	ND	ND	ND	ND
16	M	48	2022	2023	Ubiratan - PR	Araraquara-SP	Yes	No	No	No	No	DPA	Diabetic nephropathy	friend	**40**	0.2	0.2	N	**80**	0.2	0.1	N
17	F	31	2022	2022	Guarulhos - SP	Guarulhos - SP	No	Yes	No	No	No	HD	Benign nephrosclerosis	MI	**40**	0.4	0.0	N	N	0.5	0.1	N
18	M	45	2022	2022	Sao Paulo - SP	Sao Paulo - SP	No	No	No	No	No	No	Autosomal dominant polycystic kidney disease	wife	N	0.4	0.1	N	N	0.3	0.2	N
19	F	59	2023	2023	Araraquara - SP	Araraquara - SP	Yes	No	No	No	No	HD	Polycystic kidney	daughter	**40**	0.5	0.1	N	**320**	0.5	0.0	**P**
20	M	63	2023	2023	Sao Paulo - SP	Sao Paulo - SP	No	No	No	No	No	HD	Polycystic kidney disease	friend	**40**	0.5	0.1	**P**	**40**	0.2	0.1	N
21	M	64	2023	2023	Natividade da Serra - SP	Natividade da Serra - SP	Yes	No	No	No	No	HD	Not classified	son	N	0.1	0.0	N	**40**	0.1	0.0	N
22	M	45	2023	2023	Ibitinga - SP	Ibitinga - SP	No	No	No	No	No	HD	Focal segmental sclerosing glomerulopathy	cousin	N	0.1	0.0	N	**320**	0.3	0.1	N
23	F	48	2023	2023	Remanso - BA	Carapicuiba - SP	Yes	Yes	No	No	No	No	Focal segmental glomerulosclerosis	brother	**80**	**5.1**	0.2	N	**80**	**9.7**	0.0	N
24	M	26	2023	2023	Sao Paulo - SP	Sao Paulo - SP	No	No	No	No	No	HD	Atrophic kidney	mother	**40**	0.2	0.1	N	N	0.3	0.1	N
25	M	43	2023	2023	Sao Paulo - SP	Caraguatatuba - SP	No	Yes	No	No	No	HD	Membranoproliferative glomeruloneohritis	wife	**40**	0.3	0.1	N	N	0.2	0.1	N
26	M	37	2023	2023	Senador Firmino - MG	Senador Firmino - MG	Yes	Yes	No	No	No	HD	IgA nephropathy	sister	**40**	0.4	0.1	N	ND	ND	ND	ND
27	M	54	2023	2023	Sao Paulo - SP	Taboao da Serra - SP	No	Yes	No	No	No	HD	Unclear chronic renal failure	sister	**40**	0.3	0.1	N	N	0.2	0.0	N
28	F	36	2023	2023	Jacarei - SP	Jacarei - SP	No	No	No	No	No	DP	Membranoproliferative glomeruloneohritis	husband	N	0.3	0.1	N	ND	ND	ND	ND
29	M	32	2023	2023	Sao Bernardo do Campo - SP	Sao Bernardo do Campo - SP	No	Yes	No	No	No	HD	Chronic glomerulonephritis	brother	**40**	0.6	0.0	N	N	0.1	0.3	N
30	M	26	2023	2023	Presidente Prudente - SP	Pirapozinho- SP	No	No	No	No	No	CAPD	Not classified	mother	N	0.5	0.0	N	N	0.3	0.1	N
31	F	64	2023	2023	Conceiçao dos Ouros - MG	Sao Paulo - SP	No	No	No	No	No	HD	Crescent glomerulonephritis	other	N	0.2	0.1	N	ND	ND	ND	ND
32	F	56	2023	2023	Sao Joao de Miriti - RJ	Capao Redondo - RJ	No	Yes	No	No	No	HD	Hypertensive nephrosclerosis	friend	**40**	0.5	0.0	N	N	0.2	0.1	N
33	M	33	2023	2023	Santo Anastacio - SP	Cotia - SP	Yes	No	No	No	No	No	Alport syndrome	other	**40**	0.7	0.1	N	N	0.1	0.6	N
34	M	56	2023	2023	Quipapa - PE	Embu Guaçú - SP	Yes	Yes	No	No	No	HD	Autosomal dominant tubulo interstitial kidney disease	friend	**160**	0.4	0.2	N	ND	ND	ND	ND
35	F	40	2023	2023	Sao Paulo - SP	Sao Paulo - SP	No	No	No	No	No	DP	Hypertensive nephrosclerosis	sister	**40**	0.3	0.2	N	N	0.3	0.1	N
36	M	60	2023	2023	Machacalis - MG	Sao Paulo - SP	Yes	Yes	No	No	No	HD	Hypertensive nephrosclerosis	brother	**40**	0.6	0.9	N	N	0.3	0.1	N
37	M	41	2023	2023	Penapolis - SP	Santos - SP	Yes	No	No	No	No	No	Chronic glomerulonephritis	brother	N	0.4	0.1	N	N	0.3	0.1	N
38	M	49	2023	2023	Sete Lagoas - MG	Mogi das Cruzes - SP	No	No	No	No	No	HD	Polycystic kidney	brother-in-law	N	0.5	0.1	N	ND	ND	ND	ND
39	M	27	2023	2023	Sao Paulo - SP	MI	MI	MI	No	No	No	HD	Uncleared chronic renal failure	wife	**40**	**1.0**	0.4	N	N	**2.7**	0.1	N
40	M	54	2023	2023	Sao Paulo - SP	Ubatuba - SP	Yes	No	No	No	No	HD	Chronic glomerulonephritis	MI	**80**	0.3	0.5	N	N	0.1	0.4	N
41	M	39	2023	2023	Sao Paulo - SP	Sao Paulo - SP	No	No	No	No	No	HD	Chronic glomerulonephritis	uncle	**40**	0.5	0.1	N	**80**	0.4	0.4	N
42	M	39	2023	2023	Caçapava -SP	Caçapava -SP	No	Yes	No	No	No	HD	Vesical uretral reflux	cousin	**640**	0.8	0.0	N	N	0.4	0.1	**P**
43	M	43	2023	2023	Sao Paulo - SP	Sao Paulo - SP	No	Yes	No	No	No	No	Membranous glomerulonephritis	wife	N	0.7	0.3	N	N	0.4	0.1	N
44	F	53	2023	2023	Guaratingueta - SP	Guaratingueta - SP	No	No	No	No	No	HD	Lithium posisoning	friend	N	0.2	0.2	N	ND	ND	ND	ND
45	M	24	2023	2023	Sao Paulo - SP	Jucimeira - MT	Yes	MI	No	No	No	HD	Systemic lupus erythematosus	mother	N	0.6	0.0	N	ND	ND	ND	ND
46	M	26	2023	2023	Santa Isabel - SP	Guarulhos - SP	No	No	MI	MI	MI	DPA	Lupus nephritis	brother	N	0.7	0.1	N	**40**	0.9	0.4	N
47	M	59	2023	2024	Osasco - SP	Osasco - SP	No	No	No	No	No	HD	Not classified	brother	**80**	0.1	0.0	N	N	0.3	0.1	N
48	F	37	2023	2023	Osasco - SP	Sao Paulo - SP	No	No	No	No	No	HD	Focal segmental glomerulosclerosis	uncle	**160**	0.8	**1.1**	N	**160**	0.1	0.0	N

ID = identification number; M = male; F = female; age = in years; time = days between sample collection and transplantation; CL = cutaneous leishmaniasis; ML = mucosal leishmaniasis; VL = visceral leishmaniasis; HD - hemodialysis; DP = Peritoneal dialysis; CAPD = Continuous Outdoor Dialysis; N = negative; P = positive; Lm-ELISA = ≥ 40 positive; rLb6H-ELISA = ≥ 1 positive; rK39-ELISA = ≥ 1 positive; SP = Sao Paulo State; PR = Parana State; AL = Alagoas State; MG = Minas Gerais State; CE = Ceara State; MT = Mato Grosso State; RJ = Rio de Janeiro State; PE = Pernambuco State; MI = missing information; ND = not done.
